# Dysfunctional VLDL metabolism in MASLD

**DOI:** 10.1038/s44324-024-00018-1

**Published:** 2024-07-22

**Authors:** Urko M. Marigorta, Oscar Millet, Shelly C. Lu, José M. Mato

**Affiliations:** 1 Integrative Genomics Lab, CIC bioGUNE, Basque Research and Technology Alliance (BRTA), 48160 Derio, Spain; 2 Ikerbasque, Basque Foundation for Science, 48013 Bilbao, Spain; 3 Precision Medicine and Metabolism Lab, CIC bioGUNE, Basque Research and Technology Alliance (BRTA), CIBERehd, 48160 Derio, Spain; 4 Karsh Division of Gastroenterology and Hepatology, Cedars-Sinai Medical Center, Los Angeles, CA 90048 USA

**Keywords:** Metabolomics, Metabolomics, Hepatology, Liver diseases

## Abstract

Lipidomics has unveiled the intricate human lipidome, emphasizing the extensive diversity within lipid classes in mammalian tissues critical for cellular functions. This diversity poses a challenge in maintaining a delicate balance between adaptability to recurring physiological changes and overall stability. Metabolic Dysfunction-Associated Steatotic Liver Disease (MASLD), linked to factors such as obesity and diabetes, stems from a compromise in the structural and functional stability of the liver within the complexities of lipid metabolism. This compromise inaccurately senses an increase in energy status, such as during fasting-feeding cycles or an upsurge in lipogenesis. Serum lipidomic studies have delineated three distinct metabolic phenotypes, or “metabotypes” in MASLD. MASLD-A is characterized by lower very low-density lipoprotein (VLDL) secretion and triglyceride (TG) levels, associated with a reduced risk of cardiovascular disease (CVD). In contrast, MASLD-C exhibits increased VLDL secretion and TG levels, correlating with elevated CVD risk. An intermediate subtype, with a blend of features, is designated as the MASLD-B metabotype. In this perspective, we examine into recent findings that show the multifaceted regulation of VLDL secretion by S-adenosylmethionine, the primary cellular methyl donor. Furthermore, we explore the differential CVD and hepatic cancer risk across MASLD metabotypes and discuss the context and potential paths forward to gear the findings from genetic studies towards a better understanding of the observed heterogeneity in MASLD.

## Lipid diversity in humans

Fatty acids (FA) synthesis from not lipid sources including glucose, known as de novo lipogenesis (DNL), begins with the conversion of glucose to pyruvate through glycolysis in the cytoplasm. Pyruvate is subsequently converted into acetyl-CoA, a 2-carbon (2C) molecule, in the mitochondria, serving as the precursor for FA synthesis. Acetyl-CoA is then transported from the mitochondria to the cytoplasm, where it undergoes a series of enzymatic reactions catalyzed by FA synthase (FAS), a multifunctional cytosolic enzyme with seven distinct catalytic activities which is virtually identical in all biological systems. This enzyme sequentially adds 2C units derived from acetyl-CoA to a growing FA chain, resulting in the formation of the saturated 16C FA commonly known as palmitate (C16:0)^1^. Contrariwise, throughout evolution, multiple FA elongases and desaturases related to FAS have emerged, producing a wide array of FA^2^.

In the liver, palmitate serves as the precursor for the synthesis of a diverse assembly of saturated FA, including long-chain (18C) and very-long-chain (≥20C) molecular species like C18:0 (stearic acid), and C20:0 (arachidic acid). Additionally, the liver engages in the synthesis of monounsaturated and polyunsaturated FA (PUFA) from palmitate, spanning various types, including n-10, n-9, and n-7, as well as n-6 and n-3 types (where “n” indicates the position of the double bond from the methyl end of the FA), with the later derived from dietary precursors. This intricate synthesis process of FA relies on the coordinated action of different enzymes, specifically elongases and desaturases^2^.

Several transcription factors, including PPARα, LXR, SREBP-1, ChREBP, and MLX, intricately regulate diverse aspects of FA synthesis, encompassing the early stages of the process as well as the expression of elongases and desaturases. PPARα and LXR regulate FA by controlling the expression of transcription factors, such as SREBP-1 and ChREBP. SREBP-1 promotes the expression of genes involved in DNL, including FAS and acetyl-CoA carboxylase, thereby stimulating the early stages of FA synthesis, while ChREBP, activated by high carbohydrate intake, also enhances DNL. Furthermore, ChREBP and MLX form complexes that regulate the expression of elongases and desaturases^3,4^.

The process of FA synthesis and dietary FA uptake serves as a foundational pathway in lipid metabolism, generating precursors for the synthesis of an extensive array of essential lipid molecules (glycerolipids, phospholipids, and sphingolipids, among others) each fulfilling distinct roles crucial for cellular structure and function. This diversity underscores the pivotal role of FA in sustaining cellular structural integrity, the functionality of cellular membranes, signaling pathways, and energy storage mechanisms essential for the proper functioning of living organisms.

A query into the Human Metabolome Database (https://hmdb.ca/) returned 13908 results for triglycerides (TG), 5478 for phosphatidylcholines (PC), 5110 results for phosphatidylethanolamines (PE), 950 for sphingomyelins (SM), 422 for ceramides, and 148 for sterols^5^. This complex molecular landscape highlights the diversity of lipid composition in the human circulatory system^6–8^. Of note is the liver’s predominant role in synthesizing the majority of circulating lipids, underscoring its central position in orchestrating the complex and dynamic processes of lipid metabolism^8,9^.

## Balancing adaptability and stability in liver lipid metabolism

Conceptually, this large diversity of lipid species can be likened to the strands of a “frayed rope” gradually separating from the main body. This analogy captures a fundamental trade-off: while the fraying may weaken the structure integrity of the rope, it simultaneously enhances its versatility and adaptability. Similarly, the diverse array of lipids in the liver (FA, TG, PC, PE, sphingolipids, sterols, etc.) enables it to respond dynamically to shifting physiological demands, including energy requirements, biosynthesis of membranes, the production of lipid signaling molecules, and lipid storage and transport. However, this heightened adaptability also carries inherent risks and potential compromises, symbolized by the fraying of the rope. In the context of liver lipid metabolism, these compromises may manifest as issues such as the accumulation of excessive TG (liver steatosis), the potential harmful effects of TG accumulation (lipotoxicity), and an imbalance between reactive oxidative substances (ROS) generated during FA β-oxidation and antioxidants defenses (oxidative stress).

In essence, the liver faces the challenge of maintaining a delicate balance between adapting to recurrent metabolic and physiological changes (such as fasting-feeding cycles and rest-activity variations) and preserving its structural and functional stability throughout the complexities of lipid metabolism. Achieving and sustaining this equilibrium lacks a one-size-fits-all solution, as it hinges on a combination of genetic, nutritional, and environmental factors. Metabolic dysfunction-associated steatotic liver disease (MASLD), a name recently adopted by consensus for the disease previously known as non-alcoholic fatty liver disease (NAFLD)^10^, arises from multiple interconnected causes that are challenging to disentangle. MASLD is linked with and may be caused by factors contributing to obesity, diabetes, cholesterol issues, chronic kidney disease (CKD), and cardiovascular disease (CVD)^11^. Whether an individual will eventually develop MASLD and if it does, whether will go on to develop cirrhosis, liver failure and eventually hepatocellular carcinoma (HCC) or CVD, is not known.

This is an important question since MASLD has been on the rise in Western countries, closely associated with the increasing rates of obesity, sedentary lifestyles, and poor dietary choices^11–13^. In the United States and many European countries, MASLD has become a prevalent liver disease. The Western diet, characterized by high consumption of processed foods, sugars, and unhealthy fats, as well as other factors like the lack of exercise, are considered contributing factors. The impact of MASLD is not confined to Western countries. In India, for example, there has been a noticeable increase in the prevalence of MASLD, and it is becoming a significant public health concern. While traditionally India has had a lower prevalence compared to Western countries, the rising incidence of obesity, insulin resistance, and lifestyle changes are contributing to the increased prevalence of MASLD. Genetic factors and a transition to a more Westernized diet and sedentary lifestyle are influencing the disease’s emergence in the Indian population^12–14^.

The liver is a central hub in FA metabolism that orchestrates complex processes for energy production and lipid storage. Mitochondrial β-oxidation of FA generates energy, while simultaneously, TG are synthesized and stored within lipid droplets (LD)^15^. The continuous influx of FA is ensured by DNL and FA uptake from circulation^16^. Additionally, the liver releases very low-density lipoproteins (VLDL), crucial for transporting lipids to peripheral tissues^17–19^. Liver steatosis and dysregulation of energy metabolism occur when the acquisition of lipids, through FA uptake and DNL, exceeds their disposal through FA β-oxidation and VLDL secretion.

## Regulation of VLDL synthesis and secretion by S-adenosylmethionine

The synthesis and secretion of VLDL particles represents a dynamic anabolic response, particularly pronounced during fasting-feeding cycles. These anabolic processes involve intricate mechanisms that synthesize complex molecules from simpler ones, typically demanding an input of energy. Within hepatocytes, TG and other lipids undergo assembly to form VLDL particles through anabolic processes. These particles consist of a core made of TG and cholesterol esters, coated by a hydrophilic monolayer of phospholipids (predominantly PC), free cholesterol, and apolipoproteins (mainly APOB)^19^. The significance of PC and PE in regulating lipoprotein metabolism and energy utilization has been well-established. Dysregulated PC/PE ratios, either low or high, adversely affect VLDL secretion, impair LD size and function, and affect mitochondrial energy production, thereby promoting the onset of liver steatosis^20,21^.

**Fig. 1:**
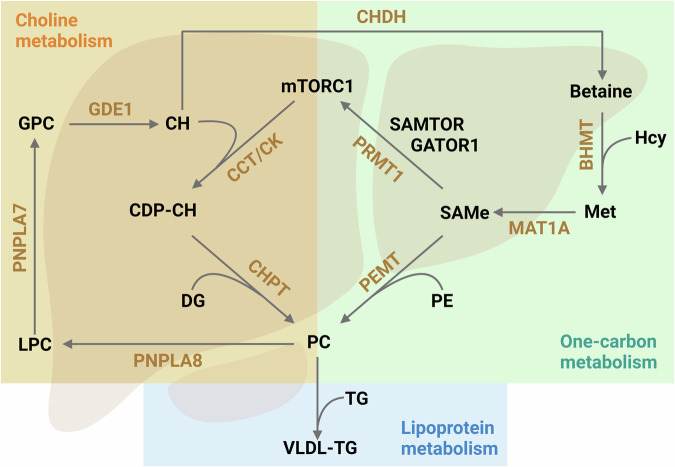
Multifaceted regulation of VLDL synthesis and export by S-adenosylmethionine. See text for a description. BHMT betaine-homocysteine methyltransferase, CCT, CTP phosphocholine cytidylyltransferase, CDP-CH CDP-choline, CH choline, CHDH choline dehydrogenase, CHPT choline phosphate transferase, CK choline kinase, DG diglycerides, GATOR1 GTPase-activating protein activity toward Rag 2, GDE1 glycerophosphocholine phosphodiesterase 1, GPC glycerophosphocholine, Hcy homocysteine, LPC lysophosphatidylcholine, MAT1A methionine adenosyltransferase 1A, Met methionine, mTORC1 mechanistic target of rapamycin kinase 1, PC phosphatidylcholine, PE phosphatidylethanolamine, PEMT phosphatidylethanolamine N-methyltransferase, PNPLA7 lysophosphatidylcholine phospholipase, PNPLA8 phosphatidylcholine phospholipase, PRMT1 protein methyltransferase 1, SAMe S-adenosylmethionine, SAMTOR SAMe sensor upstream of mTORC1, TG triglycerides, VLDL very low-density lipoproteins.

In the liver, PC is synthesized by two complementary pathways: the PE *N*-methyltransferase (PEMT) pathway and the CDP-choline (CDP-CH) pathway^21,22^. In the PEMT pathway, PC is synthesized from methionine through its conversion to S-adenosylmethionine (SAMe), followed by the methylation of PE to form PC (Fig. 1). On the other hand, the CDP-CH pathway, also known as the Kennedy pathway, utilizes choline (CH) as the initial substrate and is catalyzed by three enzymes: choline kinase, CTP: phosphocholine cytidylyltransferase (CCT), and choline phosphate transferase, with CCT, the enzyme that synthesizes CDP-CH, acting as the rate-limiting step (Fig. 1). The choline moiety of PC, through sequential deacylation by the phospholipases PNPLA8 and PNPLA7, and conversion to betaine, can be utilized to remethylate homocysteine, through a reaction catalyzed by betaine-homocysteine methyltransferase, and regenerate methionine, ultimately replenishing hepatic SAMe levels (Fig. 1)^23^.

SAMe not only regulates hepatic PC synthesis through the PEMT pathway but also exerts control over the Kennedy pathway by modulating the activity of mTORC1 (mechanistic target of rapamycin kinase 1), a key sensor of nutrient and energy status^24^. This regulation occurs via the methylation of NRPL2, the catalytic subunit of GATOR1 (GTPase-activating protein activity toward Rag 2), by protein methyltransferase 1 (PRMT1)^25^. Methylation by PRMT1 inhibits the GTPase-activating protein activity of NRPL2, resulting in mTORC1 activation^25^. Active mTORC1, in turn, stimulates PC synthesis through the CDP-CH pathway by activation of CCT, thereby stimulating the synthesis and secretion of VLDL particles (Fig. 1)^26,27^. Additionally, SAMe facilitates the methylation of NRPL2 by directly interacting with SAMTOR, prompting its dissociation from GATOR1^28^. However, SAMe binds SAMTOR with a dissociation constant of about 7 μM, which is significantly lower than the physiological concentration of SAMe in the liver, approximately 35 μM^29,30^. This raises the question about the functionality of this mechanism in the liver.

SAMe is the main biological methyl donor found across all mammalian cells, being most abundant in the adult liver. The synthesis of SAMe is carried out by the enzyme methionine adenosyltransferase (MAT). In mammals, MAT is encoded by two genes: *MAT1A*, predominantly expressed in normal liver tissue, and *MAT2A*, which is expressed in all extrahepatic tissues. Mice deficient in *Mat1a* (Mat1a-KO mice)^31^, exhibit characteristic metabolic disturbances. These include markedly reduced hepatic SAMe levels along with decreased PC levels and reduced PC to PE molar ratio, resulting in impaired VLDL secretion^31–34^. These mice develop liver steatosis spontaneously, which can progress to steatohepatitis, fibrosis, and even HCC^31–34^. Notably, patients with MASLD often exhibit diminished expression of MAT1A and low levels of hepatic SAMe^35–38^.

## Diversity in MASLD: unveiling subtypes and their lipidomic profiles

The serum lipidome encompasses a broad spectrum of lipids circulating in the bloodstream, influenced by various factors. Conditions like liver disease or metabolic disorders significantly impact hepatic lipid metabolism, consequently altering the serum lipidome. Numerous studies have sought differences in FA, TG, phospholipids, and sphingolipids to identify non-invasive serum biomarkers that differentiate between MASLD and metabolic dysfunction-associated steatohepatitis (MASH), the progressive stages previously known as non-alcoholic fatty liver (NAFL) and non-alcoholic steatohepatitis (NASH), respectively^10^. For example, Oresic et al.^39^ identified a serum lipid signature comprised of three lipids (1TG and 2 PC) for the diagnosis of MASLD in a large cohort (n = 679) using proton magnetic resonance spectroscopy to determine liver fat content. This “lipid triplet” had area under the receiver operating characteristic curve (AUROC) values of 0.74 in the discovery cohort and of 0.71 in the validation cohort^39^. Another study aiming to develop and validate a lipidomic panel to identify individuals with MASH and significant fibrosis (fibrosis stage 2 or higher)^40^, a condition referred to as at-risk MASH due to its association with elevated morbidity and mortality risk^41,42^, the authors included discovery (*n* = 790) and validation (*n* = 565) cohorts of liver biopsied individuals recruited internationally. In this study, Noureddin et al.^40^ identified 12 lipids, including 2 TG, 5 PC, 3 SM, and 1 ceramide. This panel of lipids in combination with the body mass index (BMI), ALT, and AST, showed an AUROC of 0.76 for the discovery cohort and of 0.79 for the validation cohort. Overall, these and other studies have demonstrated alterations in the levels of circulating FA, TG, phospholipids, sphingolipids, and bile acids in MASLD patients compared to subjects with normal liver. However, most studies identifying panels of potential biomarkers for differentiating between MASLD and normal liver, or between MASLD and MASH, have involved small cohorts, with few being validated or showing an AUROC ≥ 0.80 [reviewed in ref. ^8^].

The failure of these and other studies to identify lipidomic signatures accurately differentiating between individuals with MASLD and those without, as well as differentiating between the disease’s various stages (reviewed in ref. ^8^), suggests MASLD may be a heterogenous condition exhibiting different subtypes or metabotypes.

**Fig. 2:**
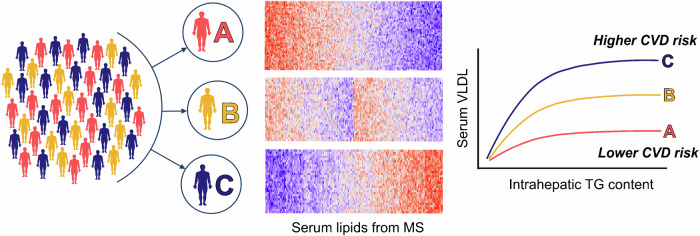
Diversity in MASLD: unveiling subtypes. Unsupervised statistical analysis classified MASLD patients into distinct subtypes or “metabotypes” based on their serum lipidomic profiles, obtained using mass spectrometry (MS), with two primary classifications: MASLD-A metabotype, characterized by lower serum VLDL levels and associated with reduced cardiovascular disease (CVD) risk; and MASLD-C metabotype, featuring increased VLDL levels and linked to elevated CVD risk. An intermediate MASLD-B metabotype was identified, showcasing a combination of features.

Lipidomics has emerged as potent tool for assessing the heterogeneity of MASLD and uncovering underlying mechanisms. Comparison of the serum lipidomic profile of Mat1a-KO mice with that of a large cohort (*n* = 535) of liver-biopsied MASLD subjects identified a subset of MASLD patients whose serum lipidome resembled that of Mat1a-KO^9^. This finding was further validated using an independent international cohort of 1154 liver biopsied MASLD patients^43^. Additionally, this study identified a panel of 11 lipids (7 PC, 2 TG, and 2 SM) that categorized MASLD into three distinct subtypes or metabotypes, each characterized by unique alterations in serum lipid homeostasis (Fig. 2). Specifically, two primary metabotypes were identified: MASLD-A metabotype, featuring lower VLDL secretion and serum TG levels, associated with a reduced CVD risk—the primary cause of mortality in MASLD^44^—; and the MASLD-C metabotype, characterized by increased VLDL secretion and serum TG levels, associated with elevated CVD risk^43^. An intermediate subtype, exhibiting a blend of features, was classified as MASLD-B metabotype. The percent of patients with MASH and fibrosis was comparable among metabotypes, although metabotypes B and C exhibited higher liver enzymes. Furthermore, the classification of MASLD into metabotypes was independent of age, sex, BMI, diabetes, or lipid lowering medication^43^.

The primary distinction between MASLD-A and MASLD-C metabotypes lies in the correlation between serum VLDL-TG concentration and intrahepatic TG content (Fig. 2)^43^. MASLD-C patients exhibit a curvilinear correlation, with serum VLDL-TG concentration saturating at steatosis grade 2 (34–66% of hepatocytes exhibiting fat accumulation). Conversely, MASLD-A patients show a serum VLDL-TG concentration independent of steatosis grade, significantly much lower than that observed in MASLD-C patients (Fig. 2). As a result, individuals with MASLD-A metabotype have reduced circulating levels of APOB containing lipoproteins, including VLDL, intermediate-density lipoproteins, and low-density lipoproteins (LDL), explaining the lower CVD risk observed in MASLD-A patients^43^. Essentially, these findings suggest that MASLD-A patients compromise the structural and functional stability of the liver by inaccurately sensing increases in its energy status, such as during an upsurge in lipogenesis, accelerating the rate of VLDL secretion.

## Differential hepatic cancer risk across MASLD subtypes

Numerous studies have highlighted, that genetic defects impairing hepatic VLDL secretion (APOB, APOC3, MTTP, TM6SF2) can lead to MASLD even in the absence of obesity or insulin resistance^45–47^. Notably, the serum metabolomic profile of Mttp-KO and Tm6sf2-KO mice closely mirrors that of MASLD-A patients^43^. These murine models of MASLD, like Mat1a-KO^34^, spontaneously develop MASLD ultimately progressing to HCC^48,49^. In contrast, the serum metabolomic profile of Ldl-receptor-KO mice, fed a high-fat/high-cholesterol diet—a mouse model of MASLD exhibiting increased VLDL secretion, liver steatohepatitis, and fibrosis without progressing to liver cancer^50^—resembles that of MASLD-C patients^43^. These findings prompt the question: can hepatic steatosis itself drive hepatic tumorigenesis in the context of impaired VLDL secretion? This is an important question, given that the prevalence of HCC in non-cirrhotic MASLD patients is estimated to be 38%, compared to 14% among non-cirrhotics with other etiologies^51^.

Recent studies show that liver deletion of *Mttp* (Mttp-LKO) increases susceptibility to diethylnitrosamine (DEN)-induced hepatic carcinogenesis in mice^52^. Intriguingly, an acceleration in liver carcinogenesis was observed in mice with simultaneous deletions of both *Mttp* and FA acid-binding protein 1 (FABP1) (Fabp1/Mttp DKO)^52^. This observation is noteworthy, especially considering that deletion of *Fabp1* (Fabp1-KO), a FA binding protein primarily involved in binding and transporting FA within hepatocytes^53^, has been shown to provide protection against diet-induced steatosis in mice^54^. DEN-treated Mttp-KO and Fabp1/Mttp DKO mice exhibit alterations in lipid metabolism (mainly TG, phospholipids, and ceramides) and energy substrate utilization when compared to control animals and show that these alterations are greater in the double KO^51^. Additionally, the double KO (Fabp1/Mttp DKO) exhibited reduced steatosis and fibrosis compared to the Mttp-KO mice^55^.

The transition from MASH to HCC is often associated with a reduction of steatosis^56^. This reduction could be due to a remodeling of hepatic metabolism, as tumor cells often require a different metabolic environment, favoring the utilization of FA as a carbon source and energy supply for tumor growth. Therefore, the mechanism by which impaired VLDL secretion promotes liver tumorigenesis might be analogous to mechanisms observed in liver metastatic tumors^57,58^. These studies have revealed that fatty liver conditions create a metastatic-friendly environment by enhancing the mobilization of LD stores, accelerating TG lipolysis, and transferring resulting FA as an energy source to neighboring tumor cells^57^. Furthermore, these studies have associated liver metastasis with heightened production of extracellular vesicles, creating an immunosuppressive tumor environment^58^. Alternatively, the reduction in FA uptake in Fabp1/Mttp DKO may result in decreased mitochondrial FA oxidation, leading to a decrease in the production of NADPH. This reduction in NADPH availability could compromise the cell’s ability to neutralize the elevated generation of ROS induced by DEN, a phenomenon associated with the development of HCC^59^. Additionally, it has also been shown, that *MAT1A* loss promotes liver cancer metastasis and sensitizes to colorectal liver metastasis^60^.

In summary, these compelling findings collectively prompt the question: Could the reduced CVD risk observed in MASLD-A metabotype patients, attributed to impaired VLDL secretion, potentially come at the expense of an elevated susceptibility to HCC? If so, to what extent are genetic factors contributing to the formation of this metabotype?

## Diversity in MASLD: genetic variants

The year 2008 marked a significant breakthrough with the discovery of the first gene variant, *PNPLA3*, strongly associated to intrahepatic TG accumulation, utilizing proton magnetic resonance spectroscopy, in a genome-wide association study (GWAS) conducted in a multiethnic population^61^. Notably, this study revealed no evidence of an association between the *PNPLA3* allele and BMI or insulin resistance, indicating a specific association between *PNPLA3* and hepatic TG content distinct from obesity or diabetes^61^. Subsequent publications not only validated this seminal study but also broadened the spectrum of genetic variants associated with this trait, confirming that MASLD is genetically influenced^62^.

These studies have uncovered a robust and reproducible association between variants in the genes *PNPLA3*, *TM6SF2*, *MBOAT7*, *HSD17B13*, and *GCKR*, and MASLD^63,64^. Furthermore, variants in *TRIB1, MTTP, GPAM, MARC1*, and *APOE* genes are also associated to MASLD^63,64^. PNPLA3, located on LD, plays a crucial role in TG and phospholipid metabolism, with its variant allele associated to altered lipid remodeling characterized by the accumulation of PUFA in diglycerides and TG, along with decreased incorporation into PC, although it does not affect VLDL secretion^65^. Interestingly, this *PNPLA3* variant is more prevalent in MASLD patients with metabotypes B and C^43^. Similarly, MBOAT7, involved in phosphatidylinositol remodeling^66^, and HSD17B13, located on LD, also impact lipid remodeling^67^. GPAM, a mitochondrial glycerol 3-phosphate acyltransferase, promotes lipid accumulation by preferring saturated FA as substrates for TG and phospholipids synthesis^68^. The E167K variant of *TM6SF2* enhances hepatic lipid accumulation by decreasing VLDL secretion, while concurrently protecting against CVD by lowering circulating lipids^69^. Other variants, such as those in TRIB1 and MTTP, influence VLDL metabolism, lowering circulating TG levels^70,71^. The P446L variant of GCKR enhances glucokinase activity, increasing glycolytic flux and TG synthesis^72^, and MARC1 encodes a mitochondrial enzyme that reduces trimethylamine N-oxide, a CVD risk factor^73^. Finally, APOE maintains lipid equilibrium in the liver by regulating the influx and efflux of lipids, with its dysregulation leading to TG accumulation^68^. It is inferred from these variants that lipid remodeling, VLDL synthesis, and export are crucial for the liver to adapt its functionality in response to changing physiological demands.

## The role of genetic heterogeneity in risk for MASLD

The collective experience from the GWAS era, including metabolic diseases, suggests that these studies have only identified a small fraction of the total number of genetic variants associated with disease susceptibility^74^. An overview of the situation with T2D illustrates this point well. As early as 2012, 63 independent signals had been associated with the disease^75^. This figure rapidly surged to the hundreds, reaching 243 loci discovered by 2018, 277 by 2022 and as many as 611 by 2024 through the recent multi-ethnic analysis conducted by the DIAMANTE consortium^76–78^. This approximately threefold increase in discovered loci every five years has been achieved through similarly large expansions in the number of cases included, which reached as many as 428,452 in the latest scan^78^. Quantitative genetic modeling indicates that the genetic architecture of most complex diseases is likely influenced by 10^4^ to 10^5^ variants spread across the genome^79,80^. Hence, an ambitious strategy focused on larger sample sizes will be needed to uncover the myriad of genes and pathways that underlie the polygenic component of MASLD.

**Fig. 3:**
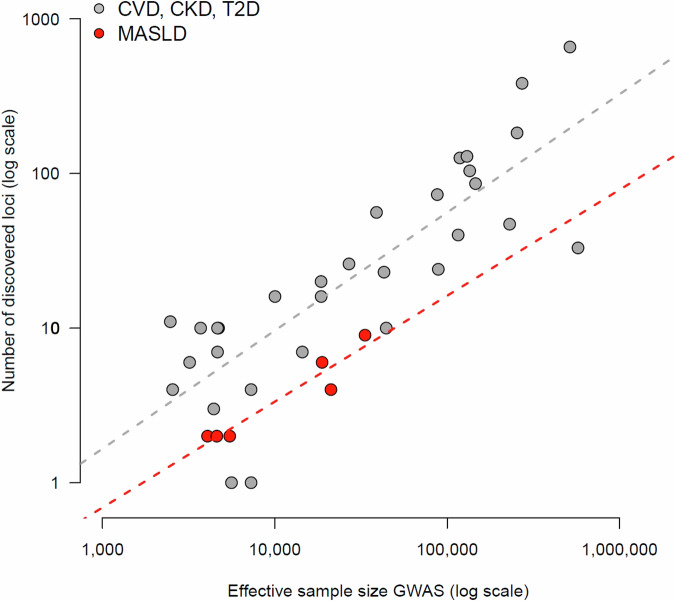
Association between sample size and GWAS discoveries in metabolic diseases. X-axis: effective sample size of GWAS studies based on the number of cases and controls used in each study. Y-axis: number of independent associations discovered in each study. Both axes are in logarithmic scale. We used the GWAS Catalog to identify studies for four metabolic diseases, namely MASLD, T2DM, CVD, and CKD. As a quality control, we selected only GWAS fulfilling four criteria, namely: i) including at least 1000 cases and 1000 controls, ii) studying individuals of a single genetic ancestry (e.g. East Asians), iii) performing association tests focused exclusively on additive effects, and iv) testing markers spread genome-wide (e.g. exome studies excluded). In total, 37 studies passed these filters: six studies for MASLD and 31 for the other three diseases (red and gray points, respectively). The dashed lines indicate the regression line for each subgroup.

Nonetheless, a comparison of the yield from GWAS suggests that the genetic architecture of MASLD presents important challenging aspects that will not be offset with brute force gained through larger sample sizes. In particular, the figure of 17 loci associated in the recent scan by Chen et al.^64^ pales clearly with the total number of genes discovered for other metabolic diseases, which in some cases such as T2DM is in the hundreds^78^. To evaluate this question, we examined the statistical delivery of GWAS for MASLD with the results for three other diseases, namely T2DM, CVD and CKD. As expected, the ability to discover new loci correlates positively with the sample size of the study (Fig. 3). However, the yield from the six selected studies for MASLD is clearly in the low range of the overall cloud of points.

Even if a qualitative approximation, this pattern suggests that the payoff of genetic studies for MASLD is notably lower. Besides the abovementioned challenges associated with phenotyping methodologies, this observation hints at a role for biological heterogeneity within disease, which is known to play an important role in determining the statistical power of genetic studies^81–83^. Although human disease genomics has focused on large-scale comparisons of cases and controls in the last two decades, increasing attention will likely pivot towards this research question. The “frayed rope” analogy discussed above also provides a useful framework to examine heterogeneity at the level of patients. Briefly, the genetic makeup of each individual does not constitute a single liability, but a range of molecular pathway-specific situations that in combination determine the risk profile and disease presentation in each person^84^. Although genetic studies have identified the backbone components of MASLD risk, new approximations are needed to characterize the hundreds of low-risk variants that underlie the versatilities in molecular profiles that are observed across MASLD patients. Notably, this new mindset has already delivered useful insights into the heterogeneity involved in MASLD susceptibility. For instance, decomposition of pleiotropic effects using the phenome-wide association study (PheWAS) framework uncovered that genetic risk for MASLD clusters into 7 mechanistic subgroups of effects^64^. In this regard, the successful characterization of the three metabotypes that emerge from liver metabolism variability demonstrates the potential of research programs focused on dissection of heterogeneity to identify subtypes within MASLD.

Taken together, the diverse array of genes and metabolomic pathways identified through GWAS underscores the intricate and multifaceted nature of the pathogenic mechanisms underlying MASLD. This complexity supports the concept that the development and progression of MASLD diverge significantly based on an individual’s unique genetic makeup. This individual variability is further manifested in the distinct lipidomic profile exhibited by each person, highlighting the intricate interplay between genetic factors and lipid metabolism pathways. For instance, variants in genes such as PNPLA3, TM6SF2, and MTTP have been associated with low VLDL secretion and the retention of TG and other lipids, leading to increased liver lipid accumulation^85,86^. On the other hand, genes like GCKR and TRIB1 contribute to MASLD by increasing glycolysis to synthesize TG via DNL^64,87^, resulting in different phenotypic expressions of MASLD. This highlights the need for a nuanced understanding of the heterogeneity within MASLD, recognizing that different combinations of genetic variants may lead to diverse metabolic outcomes and clinical presentations.

The intricate interplay between genetic factors and lipid metabolism pathways emphasizes the necessity of a personalized approach in comprehending and treating MASLD. This tailored approach allows for the development of more precise and effective therapeutic interventions that consider the unique genetic and metabolic characteristics of each individual. Therefore, the comprehensive integration of omics data—including genomics, metabolomics, and proteomics—with clinical, lifestyle, and environmental data is essential for unraveling the complexities of MASLD and advancing towards precision medicine in the field.

## References

[1] Hodson, L. & Gunn, P. J. The regulation of hepatic fatty acid synthesis and partitioning: the effect of nutritional state. *Nat. Rev. Endocrinol.* **15**, 689–700 (2019). 31554932 10.1038/s41574-019-0256-9

[2] Guillou, H., Zadravec, D., Martin, P. G. & Jacobsson, A. The key roles of elongases and desaturases in mammalian fatty acid metabolism: Insights from transgenic mice. *Prog. Lipid Res.* **49**, 186–199 (2010). 20018209 10.1016/j.plipres.2009.12.002

[3] Wang, Y. et al. Regulation of hepatic fatty acid elongase and desaturase expression in diabetes and obesity. *J. Lipid Res.* **47**, 2028–2041 (2006). 16790840 10.1194/jlr.M600177-JLR200 PMC2764365

[4] Wang, Y. et al. Transcriptional regulation of hepatic lipogenesis. *Nat. Rev. Mol. Cell Biol.* **16**, 678–689 (2015). 26490400 10.1038/nrm4074 PMC4884795

[5] Wishart, D. S. et al. HMDB 5.0: the Human Metabolome Database for 2022. *Nucleic Acids Res*. **50**, D622–D631 (2022). 34986597 10.1093/nar/gkab1062 PMC8728138

[6] Quehenberger, O. et al. Lipidomics reveals a remarkable diversity of lipids in human plasma. *J. Lipid Res.* **51**, 3299–3305 (2010). 20671299 10.1194/jlr.M009449 PMC2952570

[7] Psychogios, N. et al. The human serum metabolome. *PLoS One* **6**, e16957 (2011). 21359215 10.1371/journal.pone.0016957 PMC3040193

[8] Masoodi, M. et al. Metabolomics and lipidomics in NAFLD: biomarkers and non-invasive diagnostic tests. *Nat. Rev. Gastroenterol. Hepatol.* **18**, 835–856 (2021). 34508238 10.1038/s41575-021-00502-9

[9] Alonso, C. et al. Metabolomic Identification of Subtypes of Nonalcoholic Steatohepatitis. *Gastroenterology* **152**, 1449–1461.e7 (2017). 28132890 10.1053/j.gastro.2017.01.015 PMC5406239

[10] Rinella, M. E. et al. A multisociety Delphi consensus statement on new fatty liver disease nomenclature. *Hepatology* **78**, 1966–1986 (2023). 37363821 10.1097/HEP.0000000000000520 PMC10653297

[11] Arab, J. P., Arrese, M. & Trauner, M. Recent Insights into the Pathogenesis of Nonalcoholic Fatty Liver Disease. *Annu. Rev. Pathol.* **13**, 321–350 (2018). 29414249 10.1146/annurev-pathol-020117-043617

[12] Le, M. H. et al. Forecasted 2040 global prevalence of nonalcoholic fatty liver disease using hierarchical bayesian approach. *Clin. Mol. Hepatol.* **28**, 841–850 (2022). 36117442 10.3350/cmh.2022.0239 PMC9597215

[13] Younossi, Z. M. et al. The global epidemiology of nonalcoholic fatty liver disease (NAFLD) and nonalcoholic steatohepatitis (NASH): a systematic review. *Hepatology* **77**, 1335–1347 (2023). 36626630 10.1097/HEP.0000000000000004 PMC10026948

[14] De, A. et al. Lean Indian patients with non-alcoholic fatty liver disease (NAFLD) have less metabolic risk factors but similar liver disease severity as non-lean patients with NAFLD. *Int J. Obes.* **47**, 986–992 (2023). 10.1038/s41366-023-01346-w 37474570

[15] Zadoorian, A., Du, X. & Yang, H. Lipid droplet biogenesis and functions in health and disease. *Nat. Rev. Endocrinol.* **19**, 443–459 (2023). 37221402 10.1038/s41574-023-00845-0 PMC10204695

[16] Ipsen, D. H., Lykkesfeldt, J. & Tveden-Nyborg, P. Molecular mechanisms of hepatic lipid accumulation in non-alcoholic fatty liver disease. *Cell Mol. Life Sci.* **75**, 3313–3327 (2018). 29936596 10.1007/s00018-018-2860-6 PMC6105174

[17] Cohen, J. C., Horton, J. D. & Hobbs, H. H. Human fatty liver disease: old questions and new insights. *Science* **332**, 1519–1523 (2011). 21700865 10.1126/science.1204265 PMC3229276

[18] Mato, J. M., Alonso, C., Noureddin, M. & Lu, S. C. Biomarkers and subtypes of deranged lipid metabolism in non-alcoholic fatty liver disease. *World J. Gastroenterol.* **25**, 3009–3020 (2019). 31293337 10.3748/wjg.v25.i24.3009 PMC6603806

[19] Heeren, J. & Scheja, L. Metabolic-associated fatty liver disease and lipoprotein metabolism. *Mol. Metab.* **50**, 101238 (2021). 33892169 10.1016/j.molmet.2021.101238 PMC8324684

[20] Martínez-Uña, M. et al. Excess S-adenosylmethionine reroutes phosphatidylethanolamine towards phosphatidylcholine and triglyceride synthesis. *Hepatology* **58**, 1296–1305 (2013). 23505042 10.1002/hep.26399 PMC3720726

[21] van der Veen, J. N. et al. The critical role of phosphatidylcholine and phosphatidylethanolamine metabolism in health and disease. *Biochim. Biophys. Acta Biomembr.* **1859**, 1558–1572 (2017). 28411170 10.1016/j.bbamem.2017.04.006

[22] Vance, D. E. Phospholipid methylation in mammals: from biochemistry to physiological function. *Biochim. Biophys. Acta* **1838**, 1477–1487 (2014). 24184426 10.1016/j.bbamem.2013.10.018

[23] Hirabayashi, T. et al. Hepatic phosphatidylcholine catabolism driven by PNPLA7 and PNPLA8 supplies endogenous choline to replenish the methionine cycle with methyl groups. *Cell Rep.* **42**, 111940 (2023). 36719796 10.1016/j.celrep.2022.111940

[24] Liu, G. Y. & Sabatini, D. M. mTOR at the nexus of nutrition, growth, ageing and disease. *Nat. Rev. Mol. Cell Biol.* **21**, 183–203 (2020). 31937935 10.1038/s41580-019-0199-y PMC7102936

[25] Jiang, C. et al. PRMT1 orchestrates with SAMTOR to govern mTORC1 methionine sensing via Arg-methylation of NPRL2. *Cell Metab.* **35**, 2183–2199.e7 (2023). 38006878 10.1016/j.cmet.2023.11.001 PMC11192564

[26] Quinn, W. J. et al. mTORC1 stimulates phosphatidylcholine synthesis to promote triglyceride secretion. *J. Clin. Invest.* **127**, 4207–4215 (2017). 29035283 10.1172/JCI96036 PMC5663357

[27] Uehara, K. et al. Activation of Liver mTORC1 Protects Against NASH via Dual Regulation of VLDL-TAG Secretion and De Novo Lipogenesis. *Cell Mol. Gastroenterol. Hepatol.* **13**, 1625–1647 (2022). 35240344 10.1016/j.jcmgh.2022.02.015 PMC9046248

[28] Gu, X. et al. SAMTOR is an S-adenosylmethionine sensor for the mTORC1 pathway. *Science* **358**, 813–818 (2017). 29123071 10.1126/science.aao3265 PMC5747364

[29] Mudd, S. H. et al. Methyl balance and transmethylation fluxes in humans. *Am. J. Clin. Nutr.* **85**, 19–25 (2007). 17209172 10.1093/ajcn/85.1.19

[30] Hoffman, D. R., Marion, D. W., Cornatzer, W. E. & Duerre, J. A. S-Adenosylmethionine and S-adenosylhomocysteine metabolism in isolated rat liver. Effects of L-methionine, L-homocysteine, and adenosine. *J. Biol. Chem.* **255**, 10822–10827 (1980). 7430157

[31] Lu, S. C. et al. Methionine adenosyltransferase 1A knockout mice are predisposed to liver injury and exhibit increased expression of genes involved in proliferation. *Proc. Natl Acad. Sci. USA* **98**, 5560–5565 (2001). 11320206 10.1073/pnas.091016398 PMC33252

[32] Martínez-Chantar, M. L. et al. Spontaneous oxidative stress and liver tumors in mice lacking methionine adenosyltransferase 1A. *FASEB J.* **16**, 1292–1294 (2002). 12060674 10.1096/fj.02-0078fje

[33] Cano, A. et al. Methionine adenosyltransferase 1A gene deletion disrupts hepatic very low-density lipoprotein assembly in mice. *Hepatology* **54**, 1975–1986 (2011). 21837751 10.1002/hep.24607 PMC3222787

[34] Lu, S. C. & Mato, J. M. S-adenosylmethionine in liver health, injury, and cancer. *Physiol. Rev.* **92**, 1515–1542 (2012). 23073625 10.1152/physrev.00047.2011 PMC3698976

[35] Ahrens, M. et al. DNA methylation analysis in nonalcoholic fatty liver disease suggests distinct disease-specific and remodeling signatures after bariatric surgery. *Cell Metab.* **18**, 296–302 (2013). 23931760 10.1016/j.cmet.2013.07.004

[36] Murphy, S. K. et al. Relationship between methylome and transcriptome in patients with nonalcoholic fatty liver disease. *Gastroenterology* **145**, 1076–1087 (2013). 23916847 10.1053/j.gastro.2013.07.047 PMC3805742

[37] Moylan, C. A. et al. Hepatic gene expression profiles differentiate presymptomatic patients with mild versus severe nonalcoholic fatty liver disease. *Hepatology* **59**, 471–482 (2014). 23913408 10.1002/hep.26661 PMC3982589

[38] Guo, T. et al. S-adenosylmethionine upregulates the angiotensin receptor-binding protein ATRAP via the methylation of HuR in NAFLD. *Cell Death Dis.* **12**, 306 (2021). 33753727 10.1038/s41419-021-03591-1 PMC7985363

[39] Orešič, M. et al. Prediction of non-alcoholic fatty-liver disease and liver fat content by serum molecular lipids. *Diabetologia* **56**, 2266–2274 (2013). 23824212 10.1007/s00125-013-2981-2 PMC3764317

[40] Noureddin, M. et al. Serum identification of at-risk MASH: The metabolomics-advanced steatohepatitis fibrosis score (MASEF). *Hepatology* **79**, 135–148 (2024). 37505221 10.1097/HEP.0000000000000542 PMC10718221

[41] Hagström, H. et al. Fibrosis stage but not NASH predicts mortality and time to development of severe liver disease in biopsy-proven NAFLD. *J. Hepatol.* **67**, 1265–1273 (2017). 28803953 10.1016/j.jhep.2017.07.027

[42] Sanyal, A. J. et al. Prospective Study of Outcomes in Adults with Nonalcoholic Fatty Liver Disease. *N. Engl. J. Med.* **385**, 1559–1569 (2021). 34670043 10.1056/NEJMoa2029349 PMC8881985

[43] Martínez-Arranz, I. et al. Metabolic subtypes of patients with NAFLD exhibit distinctive cardiovascular risk profiles. *Hepatology* **76**, 1121–1134 (2022). 35220605 10.1002/hep.32427 PMC9790568

[44] Arvind, A. et al. Risk of Cardiovascular Disease in Individuals With Nonobese Nonalcoholic Fatty Liver Disease. *Hepatol. Commun.* **6**, 309–319 (2022). 34558862 10.1002/hep4.1818 PMC8793991

[45] Qin, W. et al. Missense mutation in APOC3 within the C-terminal lipid binding domain of human ApoC-III results in impaired assembly and secretion of triacylglycerol-rich very low-density lipoproteins: evidence that ApoC-III plays a major role in the formation of lipid precursors within the microsomal lumen. *J. Biol. Chem.* **286**, 27769–27780 (2011). 21676879 10.1074/jbc.M110.203679 PMC3149367

[46] Sookoian, S., Pirola, C. J., Valenti, L. & Davidson, N. O. Genetic Pathways in Nonalcoholic Fatty Liver Disease: Insights From Systems Biology. *Hepatology* **72**, 330–346 (2020). 32170962 10.1002/hep.31229 PMC7363530

[47] Romeo, S., Sanyal, A. & Valenti, L. Leveraging Human Genetics to Identify Potential New Treatments for Fatty Liver Disease. *Cell Metab.* **31**, 35–45 (2020). 31914377 10.1016/j.cmet.2019.12.002

[48] Newberry, E. P. et al. Liver-Specific Deletion of Mouse Tm6sf2 Promotes Steatosis, Fibrosis, and Hepatocellular Cancer. *Hepatology* **74**, 1203–1219 (2021). 33638902 10.1002/hep.31771 PMC8390580

[49] Newberry, E. P., Strout, G. W., Fitzpatrick, J. A. J. & Davidson, N. O. Liver-specific deletion of Mttp versus Tm6sf2 reveals distinct defects in stepwise VLDL assembly. *J. Lipid Res.* **62**, 100080 (2021). 33915141 10.1016/j.jlr.2021.100080 PMC8170145

[50] Morrison, M. C. et al. Obeticholic Acid Modulates Serum Metabolites and Gene Signatures Characteristic of Human NASH and Attenuates Inflammation and Fibrosis Progression in Ldlr-/-.Leiden Mice. *Hepatol. Commun.* **2**, 1513–1532 (2018). 30556039 10.1002/hep4.1270 PMC6287481

[51] Stine, J. G. et al. Systematic review with meta-analysis: risk of hepatocellular carcinoma in non-alcoholic steatohepatitis without cirrhosis compared to other liver diseases. *Aliment Pharm. Ther.* **48**, 696–703 (2018). 10.1111/apt.14937 PMC7495494 30136293

[52] Newberry, E. P. et al. Impaired Hepatic Very Low-Density Lipoprotein Secretion Promotes Tumorigenesis and Is Accelerated with Fabp1 Deletion. *Am. J. Pathol.*10.1016/j.ajpath.2024.02.005 (2024). 10.1016/j.ajpath.2024.02.005 PMC11156158 38417694

[53] Wang, G., Bonkovsky, H. L., de Lemos, A. & Burczynski, F. J. Recent insights into the biological functions of liver fatty acid binding protein 1. *J. Lipid Res.* **56**, 2238–2247 (2015). 26443794 10.1194/jlr.R056705 PMC4655993

[54] Newberry, E. P., Xie, Y., Kennedy, S. M., Luo, J. & Davidson, N. O. Protection against Western diet-induced obesity and hepatic steatosis in liver fatty acid-binding protein knockout mice. *Hepatology* **44**, 1191–1205 (2006). 17058218 10.1002/hep.21369

[55] Newberry, E. P. et al. Prevention of hepatic fibrosis with liver microsomal triglyceride transfer protein deletion in liver fatty acid binding protein null mice. *Hepatology* **65**, 836–852 (2017). 27862118 10.1002/hep.28941 PMC5319898

[56] Loomba, R., Friedman, S. L. & Shulman, G. I. Mechanisms and disease consequences of nonalcoholic fatty liver disease. *Cell* **184**, 2537–2564 (2021). 33989548 10.1016/j.cell.2021.04.015 PMC12168897

[57] Li, Y. et al. Hepatic lipids promote liver metastasis. *JCI Insight* **5**, e136215 (2020). 32879136 10.1172/jci.insight.136215 PMC7487169

[58] Wang, Z. et al. Extracellular vesicles in fatty liver promote a metastatic tumor microenvironment. *Cell Metab.* **35**, 1209–1226.e13 (2023). 37172577 10.1016/j.cmet.2023.04.013 PMC10524732

[59] Hayes, J. D., Dinkova-Kostova, A. T. & Tew, K. D. Oxidative Stress in Cancer. *Cancer Cell* **38**, 167–197 (2020). 32649885 10.1016/j.ccell.2020.06.001 PMC7439808

[60] Fan, W. et al. Hepatic prohibitin 1 and methionine adenosyltransferase α1 defend against primary and secondary liver cancer metastasis. *J. Hepatol.* **80**, 443–453 (2024). 38086446 10.1016/j.jhep.2023.11.022 PMC10922446

[61] Romeo, S. et al. Genetic variation in PNPLA3 confers susceptibility to nonalcoholic fatty liver disease. *Nat. Genet* **40**, 1461–1465 (2008). 18820647 10.1038/ng.257 PMC2597056

[62] Trépo, E. & Valenti, L. Update on NAFLD genetics: From new variants to the clinic. *J. Hepatol.* **72**, 1196–1209 (2020). 32145256 10.1016/j.jhep.2020.02.020

[63] Anstee, Q. M. et al. Genome-wide association study of non-alcoholic fatty liver and steatohepatitis in a histologically characterised cohort☆. *J. Hepatol.* **73**, 505–515 (2020). 32298765 10.1016/j.jhep.2020.04.003

[64] Chen, Y. et al. Genome-wide association meta-analysis identifies 17 loci associated with nonalcoholic fatty liver disease. *Nat. Genet* **55**, 1640–1650 (2023). 37709864 10.1038/s41588-023-01497-6 PMC10918428

[65] Luukkonen, P. K. et al. Human PNPLA3-I148M variant increases hepatic retention of polyunsaturated fatty acids. *JCI Insight* **4**, e127902 (2019). 31434800 10.1172/jci.insight.127902 PMC6777808

[66] Luukkonen, P. K. et al. The MBOAT7 variant rs641738 alters hepatic phosphatidylinositols and increases severity of non-alcoholic fatty liver disease in humans. *J. Hepatol.* **65**, 1263–1265 (2016). 27520876 10.1016/j.jhep.2016.07.045

[67] Luukkonen, P. K. et al. Inhibition of *HSD17B13* protects against liver fibrosis by inhibition of pyrimidine catabolism in nonalcoholic steatohepatitis. *Proc. Natl Acad. Sci. USA* **120**, e2217543120 (2023). 36669104 10.1073/pnas.2217543120 PMC9942818

[68] Jamialahmadi, O. et al. Exome-Wide Association Study on Alanine Aminotransferase Identifies Sequence Variants in the GPAM and APOE Associated With Fatty Liver Disease. *Gastroenterology* **160**, 1634–1646 (2021). 33347879 10.1053/j.gastro.2020.12.023

[69] Dongiovanni, P. et al. Transmembrane 6 superfamily member 2 gene variant disentangles nonalcoholic steatohepatitis from cardiovascular disease. *Hepatology* **61**, 506–514 (2015). 25251399 10.1002/hep.27490

[70] Burkhardt, R. et al. Trib1 is a lipid- and myocardial infarction-associated gene that regulates hepatic lipogenesis and VLDL production in mice. *J. Clin. Invest.* **120**, 4410–4414 (2010). 21084752 10.1172/JCI44213 PMC2993600

[71] Berriot-Varoqueaux, N. et al. The role of the microsomal triglygeride transfer protein in abetalipoproteinemia. *Annu. Rev. Nutr.* **20**, 663–697 (2000). 10940349 10.1146/annurev.nutr.20.1.663

[72] Beer, N. L. The P446L variant in GCKR associated with fasting plasma glucose and triglyceride levels exerts its effect through increased glucokinase activity in liver. *Hum. Mol. Genet.* **18**, 4081–1088 (2009). 19643913 10.1093/hmg/ddp357 PMC2758140

[73] Wang, Z. et al. Gut flora metabolism of phosphatidylcholine promotes cardiovascular disease. *Nature* **472**, 57–63 (2011). 21475195 10.1038/nature09922 PMC3086762

[74] Zhang, Y., Qi, G., Park, J. H. & Chatterjee, N. Estimation of complex effect-size distributions using summary-level statistics from genome-wide association studies across 32 complex traits. *Nat. Genet.* **50**, 1318–1326 (2018). 30104760 10.1038/s41588-018-0193-x

[75] Morris, A. P. et al. Large-scale association analysis provides insights into the genetic architecture and pathophysiology of type 2 diabetes. *Nat. Genet.* **44**, 981–990 (2012). 22885922 10.1038/ng.2383 PMC3442244

[76] Mahajan, A. et al. Fine-mapping type 2 diabetes loci to single-variant resolution using high-density imputation and islet-specific epigenome maps. *Nat. Genet.* **50**, 1505–1513 (2018). 30297969 10.1038/s41588-018-0241-6 PMC6287706

[77] Mahajan, A. et al. Multi-ancestry genetic study of type 2 diabetes highlights the power of diverse populations for discovery and translation. *Nat. Genet.* **54**, 560–572 (2022). 35551307 10.1038/s41588-022-01058-3 PMC9179018

[78] Suzuki, K. et al. Genetic drivers of heterogeneity in type 2 diabetes pathophysiology. *Nature* **627**, 347–357 (2024). 38374256 10.1038/s41586-024-07019-6 PMC10937372

[79] O’Connor, L. J. et al. Extreme Polygenicity of Complex Traits Is Explained by Negative Selection. *Am. J. Hum. Genet.* **105**, 456–476 (2019). 31402091 10.1016/j.ajhg.2019.07.003 PMC6732528

[80] Sinnott-Armstrong, N., Naqvi, S., Rivas, M. & Pritchard, J. K. GWAS of three molecular traits highlights core genes and pathways alongside a highly polygenic background. *Elife* **10**, e58615 (2021). 33587031 10.7554/eLife.58615 PMC7884075

[81] Marigorta, U. M. & Gibson, G. A simulation study of gene-by-environment interactions in GWAS implies ample hidden effects. *Front. Genet.* **5**, 225 (2014). 25101110 10.3389/fgene.2014.00225 PMC4104702

[82] Nagpal, S., Gibson, G. & Marigorta, U. M. Pervasive Modulation of Obesity Risk by the Environment and Genomic Background. *Genes* **9**, 411 (2018). 30110940 10.3390/genes9080411 PMC6115725

[83] Kulminski, A. M., Loika, Y., Nazarian, A. & Culminskaya, I. Quantitative and Qualitative Role of Antagonistic Heterogeneity in Genetics of Blood Lipids. *J. Gerontol. A Biol. Sci. Med. Sci.* **75**, 1811–1819 (2020). 31566214 10.1093/gerona/glz225 PMC7518561

[84] Wray, N. R., Wijmenga, C., Sullivan, P. F., Yang, J. & Visscher, P. M. Common Disease Is More Complex Than Implied by the Core Gene Omnigenic Model. *Cell* **173**, 1573–1580 (2018). 29906445 10.1016/j.cell.2018.05.051

[85] BasuRay, S., Wang, Y., Smagris, E., Cohen, J. C. & Hobbs, H. H. Accumulation of PNPLA3 on lipid droplets is the basis of associated hepatic steatosis. *Proc. Natl Acad. Sci. USA* **116**, 9521–9526 (2019). 31019090 10.1073/pnas.1901974116 PMC6511016

[86] Mahdessian, H. et al. TM6SF2 is a regulator of liver fat metabolism influencing triglyceride secretion and hepatic lipid droplet content. *Proc. Natl Acad. Sci. USA* **111**, 8913–8918 (2014). 24927523 10.1073/pnas.1323785111 PMC4066487

[87] Santoro, N. et al. Variant in the glucokinase regulatory protein (GCKR) gene is associated with fatty liver in obese children and adolescents. *Hepatology* **55**, 781–789 (2012). 22105854 10.1002/hep.24806 PMC3288435

